# Long Covid: Hormone Imbalances and/or Rather Complex Immune Dysregulations?

**DOI:** 10.1210/jendso/bvae043

**Published:** 2024-03-06

**Authors:** Christian A Koch

**Affiliations:** Department of Medicine, Division of Endocrinology, Diabetes, Metabolism, University of Florida, Jacksonville, FL 32209, USA

**Keywords:** SARS-CoV-2, adrenal, autopsy, pituitary, cortisol, immune, growth hormone

Approximately 5% of all individuals infected with severe acute respiratory syndrome coronavirus 2 (SARS-CoV-2) do not fully recover but develop long-term complications, called long Covid. SARS-CoV-2 can enter host cells via the angiotensin 2-converting enzyme receptor and the transmembrane protease serine subtype 2 and replication-competent SARS-CoV-2 can not only travel in blood during Covid-19 but can seed tissue throughout the body [1].

Autopsy studies of 19 patients with fatal Covid-19 showed that SARS-CoV-2 can induce adrenalitis [2]. Paul et al [2] did not find direct hypothalamic or pituitary effects of SARS-CoV-2 on their histomorphological and immunohistochemical analysis of 19 postmortem specimens from patients with Covid-19. Other adrenal autopsy studies during the early Covid-19 pandemic showed microscopic lesions in up to 50% of patients, including ischemic necrosis, cortical lipid degeneration, hemorrhage, focal adrenalitis, and vascular thrombosis. Stored samples of plasma collected 1 or 2 days before death of such individuals showed cortisol levels greater than 10 µg/dL but one has to keep in mind that more than 90% of circulating cortisol in serum is protein-bound and in critical illness serum total cortisol concentrations can be significantly altered.

Interestingly, a healthy 31-year-old man who received the BNT162b2 SARS-CoV-2 mRNA vaccine was reported to have developed central adrenal insufficiency with undetectable adrenocorticotropin (ACTH) levels and a serum cortisol of 1.6 µg/dL after the second vaccine injection, hypothesizing that adjuvants in the mRNA vaccine could have been the trigger [3].

In a retrospective observational study of 33 patients with Covid-19, imaging with ^18^F-FDG PET/CT showed that adrenal glands in Covid-19 patients showed lower SUVmax and SUVmean at baseline than control subjects without normalization after clinical recovery, possibly suggesting chronic adrenal hypofunction [4].

KGR and colleagues [5] assessed changes in the hypothalamo–pituitary–adrenal axis in 66 patients with mild, moderate, and severe Covid-19 at >3 months after acute infection using a 1 µg short Synacthen test. Nine of 66 subjects had a peak serum cortisol <18 µg/dL and peak serum cortisol concentrations did not differ across disease severity. Six of the 9 subjects diagnosed with adrenal insufficiency followed up at 12 months and tested then normal. A prospective study on endocrine function in 84 patients with long Covid symptoms showed that all had normal adrenal reserve as assessed by a 250 µg ACTH stimulation test. The most commonly (89%) reported symptom was fatigue and there was no association between the patients’ symptoms and hormone levels [6]. In that study abnormal adrenal function was defined as a morning serum cortisol <10 µg/dL or peak serum cortisol <18 µg/dL while using the Roche Elecsys Cortisol II monoclonal antibody assay, which by some experts is viewed to allow for lower serum cortisol cutoff values to avoid overdiagnosis of adrenal insufficiency. Long Covid symptoms included mental and/or physical fatigue, arthralgias, abdominal pain, confusion/brain fog, and others, overall representing a postexertional exhaustion reminiscent of other postviral illnesses including myalgic encephalomyelitis–chronic fatigue syndrome with suspected latent viral reactivation (cytomegalievirus, Epstein–Barr virus, herpes type 6, and herpes zoster).

Ach and colleagues [7] had the idea that SARS-CoV-2 persistence in the pituitary gland could lead to hormone deficiencies that contribute to long Covid symptoms which formed the basis of their study including 64 patients who were at least 3 months after Covid-19 infection but less than 15 months after infection. Group 1 consisted of 32 patients who were fully recovered and group 2 included 32 long Covid subjects. Hormones were evaluated by the Beckman Coulter radioimmunoassay kit. For hormone stimulation, the gold standard insulin tolerance hypoglycemia test was utilized and the authors used a low cutoff for growth hormone (GH), 3 ng/mL, diagnosing 29 of the 64 patients as GH deficient, 10 patients from group 1 and 19 patients from the long Covid group 2. Regarding the hypothalamo–pituitary–adrenal axis, 9 of the 64 subjects were found to be central, secondary adrenal insufficient with 2 patients from the Covid-19 “recovered” group 1 and 9 patients from the long Covid group 2. Stringent definition criteria for adrenal insufficiency were serum cortisol <18 µg and plasma ACTH <50 pg/mL. Of note, steroids were more frequently prescribed in group 1 patients who had more frequent hospitalizations than group 2 patients with long Covid who more frequently received the Covid-19 vaccine but had more frequent SARS-CoV-2 infection episodes. Not all patients were vaccinated and if they did get vaccinated, most patients received the Pfizer vaccine. The catalogue of long Covid symptoms as depicted in Fig. 1 by Ach and colleagues [7] is similar to the one by Mourelatos et al [6]. Ach et al [7] conclude that managing patients with long Covid should include adrenal and GH evaluation but do not provide data on how their patients’ long Covid symptoms have changed after hormone replacement therapy.

A Swiss team recently discovered that the severity of long Covid symptoms is associated with cytomegalovirus reactivation and that patients with long Covid exhibit increased complement activation at 6-month follow-up. SARS-CoV-2 may not only be considered as an infectious agent but rather as a “reservoir of replicated peptide motifs that are capable of drastic immune amplification via self-assembly with pathogen associated molecular patterns which can lead to reassembling with dsRNA into a form of proinflammatory nanocrystalline condensed matter, resulting in cooperative, multivalent immune recognition and grossly amplified inflammatory responses,” as recently proposed by a Californian team [8].

Long Covid chronic fatigue may represent a persistent low-grade endothelialitis with misaligned complex immune responses and subsequent multiorgan mitochondrial dysfunction. Monoclonal antibody infusion (MAI) with casirivimab/imdevimab antibody cocktail (Regeneron) in 3 patients with long Covid symptoms resulting from pre-Dela SARS-CoV-2 variant infection led to complete and sustained remission 5 to 7 days after MAI regardless of duration of long Covid [9]. The authors [9] proposed that MAI can (1) directly neutralize persistent SARS-CoV-2 harboring in host tissues not sampled or detected by routine testing, allowing the host restore overall immune homeostasis, (2) displace autoantibodies attached to Fc receptors, facilitating clearance and removal of those factors, and (3) the infused monoclonal antibody cocktail eliminates residual viral particles or virus-infected cells, including reactivation viruses such as Epstein–Barr virus.

## Disclosures

Prof. Koch has no conflict of interest to declare/disclose. Prof. Koch serves as an Editorial Board member of JES and Endocrinology and as an Associate Editor of the *Journal of Clinical and Translational Endocrinology*.

## Abbreviations

ACTH: adrenocorticotropin
GH: growth hormone
MAI: Monoclonal antibody infusion
SARS-CoV-2: severe acute respiratory syndrome coronavirus 2
